# Use of Body Armor by EMS Clinicians, Workplace Violence, and Racial and Ethnic Disparities in Care

**DOI:** 10.1001/jamanetworkopen.2024.56528

**Published:** 2025-01-29

**Authors:** Sarayna S. McGuire, Fernanda Bellolio, Matthew D. Sztajnkrycer, Michael J. Sveen, Chad P. Liedl, Aidan F. Mullan, Casey M. Clements

**Affiliations:** 1 Division of Prehospital Medicine, Department of Emergency Medicine, Mayo Clinic, Rochester, Minnesota; 2 Department of Emergency Medicine, Mayo Clinic, Rochester, Minnesota; 3 Mayo Clinic Ambulance Service, Mayo Clinic, Minnesota/Wisconsin; 4 Department of Quantitative Health Sciences, Mayo Clinic, Rochester, Minnesota

## Abstract

**Importance:**

There is a clear benefit to body armor against firearms; however, it remains unclear how these vests may influence day-to-day patient encounters when worn by emergency medical services (EMS).

**Objective:**

To determine the association of ballistic vests worn by EMS clinicians with workplace violence (WPV) and disparities in care among racial and/or ethnic minority patients.

**Design, Setting, and Participants:**

Prospective cohort study of a volunteer-based sample of EMS clinicians at a large, multistate EMS agency encompassing 15 ground sites across the Midwest from April 1, 2023, to March 31, 2024. Data were analyzed from May to June 2024.

**Exposure:**

External ballistic armor being used by a group of self-selected clinicians on every run.

**Main Outcomes and Measures:**

Prevalence and characteristics associated with WPV and with declines of treatment and/or transport compared between crews with 1 or more vested vs no vested members.

**Results:**

A total of 156 of 415 staff (37.6%) opted in to wear the vests, including 77 male participants (49.4%). Prevalence of WPV was higher for vested crews (1.11 vs 0.85 cases per 100 runs; adjusted risk ratio [aRR], 1.28; 95% CI, 1.10 to 1.50; *P* = .001) and was due to higher rates of verbal abuse. The presence of 1 or more vested crew members increased the likelihood of all patients declining EMS treatment and/or transport; however, effect size was highest among patients with an unknown race and/or ethnicity (2234 [21.1%] vs 2134 [16.5%] patients; aRR, 1.19; 95% CI, 1.10 to 1.27; *P* < .001), followed by racial and/or ethnic minority patients (708 [16.7%] vs 399 [13.8%] patients; aRR, 1.18; 95% CI, 1.05 to 1.33; *P* = .01). Analyses of individual minority groups revealed a significant increase only in Black or African American patients declining treatment and/or transport by vested crews (461 [17.6%] vs 223 [13.7%] patients; RR, 1.28; 95% CI, 1.10 to 1.49; *P* = .002).

**Conclusions and Relevance:**

In this cohort study, vested crews experienced increased prevalence of WPV compared with nonvested crews. Use of vests increased the frequency of all patients declining EMS treatment and/or transport. Among minority groups, there was a significant increase in Black or African American patients declining treatment and/or transport. Agencies should consider benefits and unintended consequences of EMS clinicians wearing body armor.

## Key Points

**Question:**

What is the association between body armor (ballistic vests) worn by emergency medical services (EMS) clinicians with workplace violence (WPV) and racial disparities in care?

**Findings:**

In this cohort study involving 415 EMS staff members, prevalence of WPV was significantly higher for vested crews (1.11 vs 0.85 cases per 100 runs; *P* = .001). Presence of 1 or more vested crew members increased the likelihood of all patients declining EMS treatment and/or transport with effect size highest among patients with an unknown race and/or ethnicity followed by racial and/or ethnic minority patients.

**Meaning:**

These findings suggest that agencies should consider benefits and unintended consequences of EMS clinicians wearing body armor.

## Teaser

This cohort study evaluates whether the use of ballistic vests by emergency medical services (EMS) clinicians is associated with rates of workplace violence or with disparities in care by race and ethnicity.

## Introduction

Workplace violence (WPV) is a significant occupational hazard, with data from the Bureau of Labor Statistics (BLS) demonstrating the rate of WPV injuries among health care practitioners to be more than 3 times higher than the national average for all occupations in the US.^1^ Included within this statistic are emergency medical services (EMS) personnel who are commonly exposed to WPV in the field and in the homes of patients, often with fewer resources and staff to mitigate violence.^2,3,4,5,6,7,8^ Among the types of WPV experienced by EMS clinicians is the risk of aggravated assault with a deadly weapon, with a prior survey study of EMS clinicians revealing 2% had experienced an actual or attempted stabbing and 1% had experienced an actual or attempted shooting in a 12-month period.^7^

Given concerns for safety, EMS agencies are seeking novel ways to protect staff, including the use of external body armor vests, which can be categorized as being ballistic-resistant, stab-resistant, or both.^9^ Aside from use by the military on the battlefield, body armor has historically been used to protect law enforcement (LE) and correctional officers.^9^ Agency policies have differed regarding whether vests should only be worn when responding to certain high-risk calls (eg, shootings, stabbings, and so forth),^10^ or on every patient encounter (run).^11,12^ Additionally, agencies must consider whether to purchase external vs concealable vests, with a major concern of the former being that they may lead to EMS clinicians becoming targets for violence, potentially due to being confused for LE.^13^ Confusion between EMS and LE by the public is longstanding,^13,14,15^ and with increasing violence against LE,^16,17^ EMS agencies are becoming more cognizant regarding their shared appearance, leading some to even modify their uniform to better distinguish their staff.^18,19^

There is a clear benefit to body armor against firearms, with over 3100 LE officers saved from death or serious injury since 1987.^20,21^ Additionally, body armor provides the wearer with some degree of protection against blunt trauma inflicted to the torso where it is worn. However, for clinicians wearing external body armor, it remains unclear how these vests influence day-to-day WPV not involving the penetrating weapons they were designed for. There exists a possibility that sharing a similar appearance to LE may lead to EMS being treated like LE by the public, with an unintended increase in hostility and WPV overall. Furthermore, published perspectives from Black and Hispanic patients have indicated that LE presence in health care may be distressing for patients who do not trust the police or have had negative experiences with them in the past.^22,23,24^ To our knowledge, prior literature has not assessed outcomes of external vest use among EMS clinicians and influences on patient care.

Our primary objective was to determine whether EMS clinicians wearing external ballistic vests experienced an increase in WPV compared with their nonvested colleagues. Our secondary objective was to assess for potential disparities in the care of racial and ethnic minority patients when being cared for by vested vs nonvested clinicians.

## Methods

### Study Design and Setting

This was a prospective cohort study from April 1, 2023, to March 31, 2024, within a large, multistate EMS agency encompassing 15 ground sites across the Midwest with approximately 415 ground-based staff employed at time of the study. The agency averages 122 000 ground calls for service annually, including 96 000 calls to 911. We included all 911 calls, including priority 1 (emergent, hereafter referred to as P1) and priority 2 (urgent, hereafter referred to as P2) runs and excluded interfacility transports. The study was reviewed by the institutional review board at the Mayo Clinic and was granted an exemption under applicable federal regulations, indicating that a formal consent process was not required. Strengthening the Reporting of Observational Studies in Epidemiology (STROBE) reporting guidelines were followed.^25^

### Data Sources

The agency uses a checkbox embedded within the prehospital electronic medical record (EMR), Zoll RescueNet ePCR software (Zoll Medical Corp), asking EMS clinicians to indicate at time of run documentation whether any WPV was experienced during the run. Selecting an option (yes, verbal; yes, physical; yes, both verbal and physical; or no) is required to close out the call for most runs, with the exception of certain calls, such as those canceled or aborted or where no patient contact was made. The Joint Commission’s definitions^26^ of verbal abuse and physical assault were disseminated to staff before and throughout the study period. Within the EMR, the categorization of patient race and ethnicity is a combined feature and documentation is encouraged but optional for EMS clinicians.

### Intervention

Agency leadership purchased National Institute of Justice (NIJ) level II ballistic armor with an external black Traverse-slick carrier for interested staff (Figure). EMS clinicians who opted in to the intervention contractually agreed to wear their custom-fitted vest on every 911 (P1 or P2) call for a period of 5 years with compliance enforced by agency leadership. NIJ level II ballistic armor is rated to stop 9 mm and 0.44 Magnum ammunition fired from short-barrel handguns and offers no rifle protection. Vests also include front and rear titanium plate inserts designed to stop edged weapons (eg, knives or scissors). The titanium plates provide additional blunt force trauma protection but do not provide additional ballistic protection.^27^ Additionally, the agency purchased 2 one-size-fits-most rifle plate carriers with front and back steel NIJ level III plus plates for each ambulance to be used in place of the soft armor in the event of an active shooter.^27^ The decision to acquire ballistic vests was made by agency leadership and unrelated to this research study, which was later developed to study the planned intervention. Agency leadership pursued acquisition of ballistic armor for staff as a proactive approach with no incidents of penetrating violence against staff in the years leading up to vest acquisition. Following sessions for individual sizing, vests were delivered to agency sites beginning the week of March 13, 2023.

**Figure.**
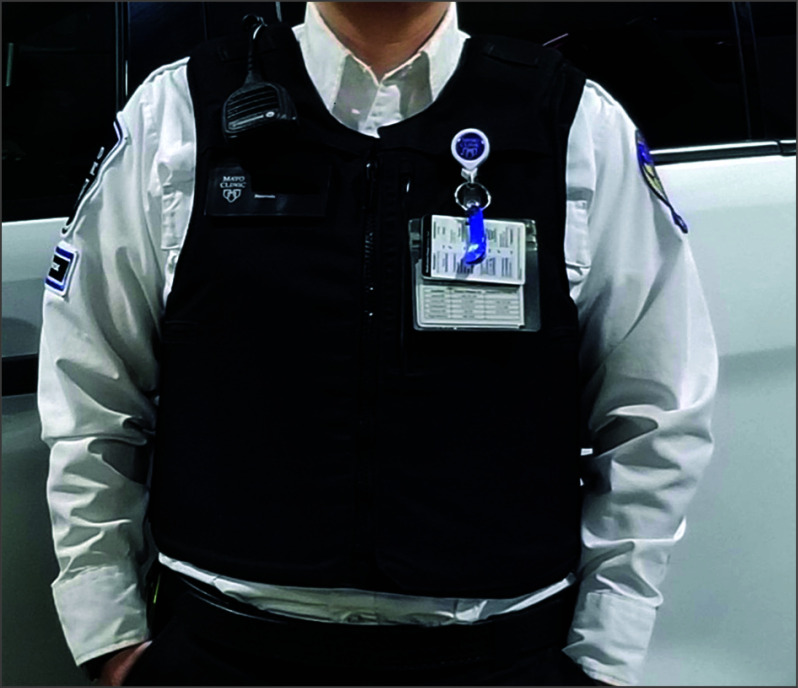
Appearance of Emergency Medical Services Agency Uniform With National Institute of Justice Level II Ballistic Armor, External Traverse-Slick Carrier With Front and Back Titanium Plates Used with permission of Mayo Foundation for Medical Education and Research.

### Variables

EMS clinician gender, certification, and years at the agency were recorded. Advanced crew certifications included physicians, 1 physician assistant, and critical care registered nurses. Crew composition and vest use of all members were recorded for each run. Run priority was tracked, and WPV was documented for each encounter as described in the prior section. Patient age, race and ethnicity, and chief concern (CC) were recorded. EMS clinician documentation of primary impression and perceived barriers to care on the run were tracked. Within our EMS agency’s EMR, patient race and ethnicity represent a combined category and EMS clinicians are asked to inquire from their patients their preferred race/ethnicity for documentation purposes. Race and ethnicity were assessed to evaluate disparities in care. Racial and ethnic minority patients included those documented as American Indian or Alaska Native, Asian, Black or African American, Hispanic or Latino, or Native Hawaiian or Pacific Islander; patients who identified as non-Hispanic White were classified as such. Declining treatment and/or transport by EMS clinicians was defined as EMS runs with documented encounter outcomes of 1 of the following: refusal of treatment and transport against medical advice (AMA), no treatment, treatment but no transport, or transport by another method (not ambulance).

### Outcomes

The primary outcome was the overall prevalence of WPV experienced by EMS crews with 1 or more vested crew member compared with those with no vested crew members. The secondary outcome was assessment for potential disparities in care of racial and ethnic minority patients when being cared for by vested vs nonvested EMS clinicians, determined by rate of patients declining EMS treatment and/or transport.

### Statistical Analysis

Numeric characteristics were summarized with medians and IQRs; categorical characteristics were summarized with frequency counts and percentages. The prevalence of WPV was calculated as the number of EMS runs in which WPV occurred per 100 runs. Differences in the prevalence of WPV between runs in which no EMS crew member wore a ballistic vest against runs in which at least 1 crew member wore a vest were summarized with risk differences (RDs) and 95% CIs. The prevalence of WPV was also compared between runs with no vested EMS crew member and at least 1 vested crew member using population-averaged modified Poisson generalized estimating equations (GEEs). The benefit to using GEEs over standard regression is that GEEs allow for the individual EMS encounters to be clustered, accounting for multiple encounters handled by the same EMS crew, which would break a primary assumption of regression. Encounters handled by the same crew were assumed to have constant correlation, and SEs were estimated using the Eicker-Huber-White sandwich estimator. These GEEs were both unadjusted and adjusted for patient age, race and ethnicity, CC, run priority level, EMS crew primary impression, crew size, crew gender, average crew member years of experience, and patient substance use listed as a barrier to treatment. Model results were reported as relative risks (RRs) and adjusted RRs (aRRs) with 95% CIs.

To determine whether staff opting in to the vests were more likely to experience WPV at baseline (self-selection bias), we evaluated WPV in preintervention runs (November 14, 2022 to March 13, 2023). This date range was chosen based on the WPV checkbox being implemented across the agency on November 14, 2022, and vest distribution beginning on March 13, 2023.

The proportion of patients declining transport was compared between EMS crews with 1 or more vested member vs no vested members using modified Poisson GEEs accounting for EMS crews responsible for multiple encounters. GEEs were unadjusted and adjusted for patient age, CC, run priority level, EMS crew primary impression, crew size, crew gender, average crew member years of experience, and patient substance use. Subgroup analysis divided the patients by race and ethnicity (non-Hispanic White, racial or ethnic minority, and unknown race) to assess whether specific demographic groups were more likely to decline treatment and/or transport in the presence of EMS crews wearing ballistic vests. Results were reported as RRs with 95% CIs. Further analysis among specific racial and ethnic minority groups was performed using only unadjusted GEEs due to sample size limitations. All tests were 2-sided, and *P* values less than .05 were considered significant. Analysis was conducted using R version 4.2.2 (R Project for Statistical Computing). Data were analyzed from May to June 2024.

## Results

### Characteristics of EMS Crews and Overall WPV

A total of 156 of 415 staff members (37.6%) opted in to wear the vests during the study period, including 77 male participants (49.4%). Between April 1, 2023, and March 31, 2024, there were 93 855 P1 and P2 runs. After exclusion of 17 705 runs (18.9%) in which crews were canceled en route or no patient contact was made and 204 (0.22%) runs in which the WPV checkbox was not used, 75 946 runs remained and were included in final analysis. A total of 752 (0.99 per 100; 95% CI, 0.92 to 1.06) runs were identified by EMS clinicians as having WPV, including 616 with verbal abuse, 337 with physical assault, and 201 with both verbal and physical violence. There were no incidents of violence inflicted against EMS clinicians with a penetrating object and no incidents in which a rifle plate carrier was used during a run in which patient contact was made. There were no patient encounters involving trauma secondary to gunshot wounds or stabbings during the study period.

The majority of runs (66 080 runs [87.0%]) consisted of 2-member all-paramedic (46 441 runs [61.2%]) crews (Table 1). EMS crew number of years of employment at the agency was measured as the mean years for all members of the crew. Among all crews that experienced violent encounters, the median (IQR) number of years employed was 4.6 (2.5-8.4) years, compared with 5.7 (3.1-9.8) years on nonviolent encounters (*P* < .001). Unknown gender composition (129 [17.2%] WPV vs 10 323 [13.7%] non-WPV) and all-female crews (97 [12.9%] WPV vs 8092 [10.8%] non-WPV) were found to have a higher prevalence of WPV compared with mixed-gender (293 [39.0%] WPV vs 29 487 [39.2%] non-WPV) or all-male crews (233 [31.0%] WPV vs 27 292 [36.3%] non-WPV) (*P* = .002).

**Table 1.** Demographics and Characteristics of Emergency Medical Services (EMS) Crews on Runs During Study Period

Characteristics	Crew Members, No. (%)			*P* value
	All runs (N = 75 946)	No violence (n = 75 194)	Violence (n = 752)	
Total No. of crew members				
1	474 (0.6)	469 (0.6)	5 (0.7)	.79
2	66 080 (87.0)	65 420 (87.0)	660 (87.8)	
3	9392 (12.4)	9305 (12.4)	87 (11.6)	
Crew certifications				.49
All paramedics	46 441 (61.2)	46 005 (61.2)	436 (58.0)	
Paramedics and EMTs	25 818 (34.0)	25 540 (34.0)	278 (37.0)	
All EMTs	2649 (3.5)	2622 (3.5)	27 (3.6)	
Any advanced certification	429 (0.6)	425 (0.6)	4 (0.5)	
Unknown certifications	609 (0.8)	602 (0.8)	7 (0.9)	
Crew gender				
All female	8189 (10.8)	8092 (10.8)	97 (12.9)	.002
Mixed gender	29 780 (39.2)	29 487 (39.2)	293 (39.0)	
All male	27 525 (36.2)	27 292 (36.3)	233 (31.0)	
Unknown	10 452 (13.8)	10 323 (13.7)	129 (17.2)	
Protective vest use				
No members vested	34 897 (45.9)	34 599 (46.0)	298 (39.6)	.002
Some members vested	32 522 (42.8)	32 164 (42.8)	358 (47.6)	
All members vested	8527 (11.2)	8431 (11.2)	96 (12.8)	
Crew y at agency, median (IQR)				
Mean	5.7 (3.0-9.8)	5.7 (3.1-9.8)	4.6 (2.5-8.4)	<.001
Maximum	7.7 (4.1-14.5)	7.7 (4.1-14.5)	6.4 (3.4-11.3)	<.001

Abbreviation: EMT, emergency medical technician.

### Characteristics of Patients and EMS Runs

WPV was more likely in runs with younger patients with a median (IQR) of 44 (32-62) years vs 63 (41-77) years among non-WPV runs (RR per 5 years, 0.91; 95% CI, 0.89-0.92; *P* < .001) (Table 2). Among violent runs, patient CC and crew primary impression were documented as behavioral in nearly half (334 [44.4%] CC and 375 [49.9%] impressions), compared with only 4345 (5.8%) CC (RR, 7.69; 95% CI, 7.06-8.37; *P* = .001) and 5963 (7.9%) impressions (RR, 6.29; 95% CI, 5.83-6.78; *P* < .001) of non-WPV runs. Perceived barriers to treatment included physical impairment (103 [13.7%] WPV vs 2814 [3.7%] non-WPV; RR, 3.66; 95% CI, 3.05-4.40; *P* < .001), suspected intoxication (130 [17.3%] WPV vs 750 [1.0%] non-WPV; RR, 17.33; 95% CI, 14.60-20.59; *P* < .001), uncooperative patient (260 [34.6%] WPV vs 617 [0.8%] non-WPV; RR, 42.14; 95% CI, 37.15-47.79; *P* < .001), emotional distress (105 [14.0%] WPV vs 460 [0.6%] non-WPV; RR, 22.82; 95% CI, 18.70-27.86; *P* < .001), and physical restraints placed by either EMS or LE (61 [8.1%] WPV vs 190 [0.3%] non-WPV; RR, 32.10; 95% CI, 24.28-42.45; *P* < .001). Crews spent a longer time on scene during runs with WPV with a median (IQR) of 17.0 (11.2-25.0) minutes, compared with 14.5 (10.2-19.9) minutes on runs without WPV (RR per 10 minutes, 1.03; 95% CI, 1.02-1.04; *P* < .001). Increased violence occurred on runs in which patients ultimately refused care AMA (32 [4.3%] WPV vs 977 [1.3%] non-WPV; RR, 3.28; 95% CI, 2.32-4.62; *P* < .001).

**Table 2.** Demographics and Clinical Characteristics of Patients and Emergency Medical Services (EMS) Runs During Study Period

Characteristics	Patients, No. (%)			*P* value
	All runs (N = 75 946)	No violence (n = 75 194)	Violence (n = 752)	
Age, median (IQR), y	63 (41-77)	63 (41-77)	44 (32-62)	<.001
Race and ethnicity				
American Indian or Alaska Native	871 (1.1)	843 (1.1)	28 (3.7)	<.001
Asian	512 (0.7)	507 (0.7)	5 (0.7)	
Black or African American	4244 (5.6)	4191 (5.6)	53 (7.0)	
Hispanic or Latino	1358 (1.8)	1333 (1.8)	25 (3.3)	
Native Hawaiian or Pacific Islander	142 (0.2)	140 (0.2)	2 (0.3)	
Non-Hispanic White	45 313 (59.7)	44 888 (59.7)	425 (56.5)	
Unknown race and ethnicity	23 506 (31.0)	23 292 (31.0)	214 (28.5)	
Chief concern				
Medical	42 712 (56.2)	42 456 (56.5)	256 (34.0)	.001
Trauma	11 244 (14.8)	11 149 (14.8)	95 (12.6)	
Respiratory	6656 (8.8)	6628 (8.8)	28 (3.7)	
Cardiac	5438 (7.2)	5428 (7.2)	10 (1.3)	
Behavioral	4679 (6.2)	4345 (5.8)	334 (44.4)	
Other	776 (1.0)	775 (1.0)	1 (0.1)	
Unknown	4441 (5.8)	4413 (5.9)	28 (3.7)	
Primary impression				
Medical	42 264 (55.7)	42 014 (55.9)	250 (33.2)	<.001
Trauma	10 740 (14.1)	10 662 (14.2)	78 (10.4)	
Respiratory	4523 (6.0)	4513 (6.0)	10 (1.3)	
Cardiac	5430 (7.1)	5423 (7.2)	7 (0.9)	
Behavioral	6338 (8.3)	5963 (7.9)	375 (49.9)	
Scheduled transfer	487 (0.6)	485 (0.6)	2 (0.3)	
Other	1690 (2.2)	1684 (2.2)	6 (0.8)	
Unknown	4474 (5.9)	4450 (5.9)	24 (3.2)	
Barriers to treatment				
Physical impairment	2917 (3.8)	2814 (3.7)	103 (13.7)	<.001
Suspected substance abuse	880 (1.2)	750 (1.0)	130 (17.3)	<.001
Uncooperative patient	877 (1.2)	617 (0.8)	260 (34.6)	<.001
Emotional distress	565 (0.7)	460 (0.6)	105 (14.0)	<.001
Unconscious patient	564 (0.7)	560 (0.7)	4 (0.5)	.50
Physical restraints	251 (0.3)	190 (0.3)	61 (8.1)	<.001
Other barrier	1043 (1.4)	1034 (1.4)	9 (1.2)	.68
No barriers	69 940 (92.1)	69 578 (92.5)	362 (48.1)	<.001
EMS call characteristics				
EMS run priority				
P1	53 211 (70.1)	52 753 (70.2)	458 (60.9)	<.001
P2	22 735 (29.9)	22 441 (29.8)	294 (39.1)	
Time on scene, median (IQR), min	14.5 (10.2-20.0)	14.5 (10.2-19.9)	17.0 (11.2-25.0)	<.001
Call outcome				
Treated, transported by EMS	60 722 (80.0)	60 073 (79.9)	649 (86.3)	<.001
No treatment	9490 (12.5)	9445 (12.6)	45 (6.0)	
Treated, not transported by EMS	2185 (2.9)	2168 (2.9)	17 (2.3)	
Interfacility transport	1858 (2.4)	1851 (2.5)	7 (0.9)	
AMA refusal of care	1009 (1.3)	977 (1.3)	32 (4.3)	
Discontinue resuscitation	435 (0.6)	434 (0.6)	1 (0.1)	
Scene death	166 (0.2)	165 (0.2)	1 (0.1)	
Stand by	81 (0.1)	81 (0.1)	0 (0)	

Abbreviations: AMA, against medical advice; P1, emergent; P2, urgent.

### Association of Vests and WPV

Prevalence of WPV was higher for crews wearing vests (1.11 vs 0.85 cases per 100 runs; RD, 0.25; 95% CI, 0.11 to 0.39; *P* < .001) (Table 3). After accounting for patient (age, race and ethnicity, and CC) and encounter (run priority level, EMS crew primary impression, crew size, crew gender, average crew years of experience, and patient substance use as a barrier to treatment) characteristics, EMS crews wearing 1 or more vests had a 28% higher risk for WPV compared with nonvested crews (adjusted RR, 1.28; 95% CI, 1.10 to 1.50; *P* = .001). This increase was due to higher rates of verbal abuse for vested crews (0.91 vs 0.70 cases per 100 runs; RD, 0.21; 95% CI, 0.08 to 0.33; adjusted RR, 1.31; 95% CI, 1.11 to 1.56; *P* = .001). There was no significant difference in physical assault between vested vs nonvested crews (0.48 vs 0.40 cases per 100 runs; RD, 0.08; 95% CI, −0.02 to 0.17; adjusted RR, 1.33; 95% CI, 0.90 to 1.42; *P* = .29).

**Table 3.** Prevalence of Workplace Violence Among Vested vs Unvested Emergency Medical Services Crews

Type of violence documented	Prevalence of violence per 100 runs (95% CI)		GEE			
			Unadjusted		Adjusted^a^	
	No members vested (n = 34 897)	>1 Member(s) vested (n = 41 049)	RR (95% CI)	*P* value	RR (95% CI)	*P* value
Any violence	0.85 (0.76-0.96)	1.11 (1.01-1.21)	1.31 (1.12-1.53)	<.001	1.28 (1.10-1.50)	.001
Verbal abuse	0.70 (0.61-0.79)	0.91 (0.82-1.00)	1.31 (1.10-1.55)	.002	1.31 (1.11-1.56)	.001
Physical assault	0.40 (0.34-0.47)	0.48 (0.42-0.55)	1.21 (0.97-1.52)	.096	1.13 (0.90-1.42)	.29

Abbreviations: GEE, generalized estimating equations; RR, risk ratio.

^a^ Adjusted for patient age, race and ethnicity, chief concern, run priority, crew primary impression, crew size, crew gender, average crew years of experience, and patient substance use.

### Association of Vests and Disparities of Care

Vested crews resulted in increased frequency of treatment and/or transport declines among all patients (6828 [16.6%] vs 5051 [14.5%]; adjusted RR, 1.11; 95% CI, 1.07 to 1.16; *P* < .001) (Table 4). The effect size was greatest in the unknown race and ethnicity patient group (2234 [21.1%] vs 2134 [16.5%] patients; adjusted RR, 1.19; 95% CI, 1.10 to 1.27; *P* < .001), followed by the racial and ethnic minority group (708 [16.7%] vs 399 [13.8%] patients; adjusted RR, 1.18; 95% CI, 1.05 to 1.33; *P* < .001). While the non-Hispanic White group had increased frequency of treatment and/or transport declines with vested crews (3886 [14.8%] vs 2518 [13.2%]; unadjusted RR, 1.12; 95% CI, 1.06 to 1.18; *P* < .001), this difference was not significant after accounting for other patient demographics and EMS encounter characteristics (adjusted RR, 1.05; 95% CI, 0.99 to 1.10; *P* = .09). A sensitivity analysis excluding all patients with unknown race still found a significant increase in patients declining treatment and/or transport from EMS crews wearing 1 or more vests (4594 [15.1%] vs 2917 [13.3%]; adjusted RR, 1.05; 95% CI, 1.00 to 1.10; *P* = .04). Analysis of individual patient minority race and ethnicity groups revealed a significant increase only in Black or African American patients declining treatment and/or transport by vested crews (461 [17.6%] vs 223 [13.7%] patients; unadjusted RR, 1.28; 95% CI, 1.10 to 1.49; *P* = .002) (eTable 1 in Supplement 1).

**Table 4.** Frequency of Outcomes Indicating Treatment and/or Transport Declined by Patients Based on Emergency Medical Services (EMS) Crew Wearing Vests With Unadjusted and Adjusted Risk Ratio

EMS run outcome	Patients, No. (%)		Unadjusted RR (95% CI)	*P* value	Adjusted RR (95% CI)^a^	*P* value
	No members vested	>1 Member(s) vested				
All patients						
No.	34 897	41 049	1.15 (1.11-1.20)	<.001	1.11 (1.07-1.16)	<.001
Declined treatment/transport	5051 (14.5)	6828 (16.6)				
Did not decline treatment/transport	29 846 (85.5)	34 221 (83.4)				
Racial and/or ethnic minority patients^b^						
No.	2899	4228	1.21 (1.07-1.36)	.001	1.18 (1.05-1.33)	.005
Declined treatment/transport	399 (13.8)	708 (16.7)				
Did not decline treatment/transport	2500 (86.2)	3520 (83.3)				
Non-Hispanic White patients						
No.	19 079	26 234	1.12 (1.06-1.18)	<.001	1.05 (0.99-1.10)	.091
Declined treatment/transport	2518 (13.2)	3886 (14.8)				
Did not decline treatment/transport	16 561 (86.8)	22 348 (85.2)				
Unknown race						
No.	12 919	10 587	1.27 (1.17-1.36)	<.001	1.19 (1.10-1.27)	<.001
Declined treatment/transport	2134 (16.5)	2234 (21.1)				
Did not decline treatment/transport	10 785 (83.5)	8353 (78.9)				
Patients with known race						
No.	21 978	30 462	1.13 (1.07-1.19)	<.001	1.05 (1.00-1.10)	.037
Declined treatment/transport	2917 (13.3)	4594 (15.1)				
Did not decline treatment/transport	19 061 (86.7)	25 868 (84.9)				

Abbreviation: RR, risk ratio.

^a^ Adjusted for patient age, chief concern, run priority, crew primary impression, crew size, crew gender, average crew years of experience, and patient substance use.

^b^ Includes those documented as American Indian or Alaska Native; Asian; Black or African American; Hispanic or Latino; or Native Hawaiian or Pacific Islander.

### Prevalence of WPV Before Vest Implementation

Prevalence of WPV prevest implementation was 1.12 (95% CI, 0.98 to 1.28) cases per 100 runs (eTable 2 in Supplement 1) among clinicians who opted in to the vest compared with staff who did not opt in (0.97; 95% CI, 0.89 to 1.06 cases per 100 runs). Clinicians who opted in to the vest program did not experience significantly higher rates of WPV prevest implementation than clinicians who did not opt in (RR, 1.16; 95% CI, 0.98 to 1.36; *P* = .08).

## Discussion

Acts of violence against EMS clinicians involving weapons result in significant morbidity and mortality and are receiving increasing attention nationwide.^28,29,30,31^ There are undeniable benefits of body armor in protecting wearers from the penetrating weapons they are designed for.^20,21^ However, agencies faced with the decision of acquiring body armor for their staff should consider the full spectrum of benefits and risks. In terms of the latter, this study demonstrates 2 particular risks that to our knowledge have not been demonstrated previously in the literature. EMS clinicians wearing external ballistic vests on 911 calls experienced an increase in WPV compared with their nonvested colleagues, and this violence was largely verbal. An initial presumption was that this finding may be attributable to the staff who opted in to the vest intervention experiencing increased WPV at baseline; however, analysis of WPV experienced prevest vs postvest implementation among both vested and nonvested cohorts suggests that this was not the case. Although we hypothesize this may be related to a shared appearance with that of law enforcement, we did not interview patients for this study. We recognize that crew experience, run, scene, or patient factors may be contributory. However, EMS clinician focus group participants in prior qualitative literature have referenced a desire for covert rather than external body armor to appear nonthreatening to the public, with the concern that they may present an aggressive posture by wearing readily visible armor.^32^ Future research should explore this finding further from the patient perspective to better determine the reason for the difference we observed between staff groups.

Our finding of increased treatment and/or transport declines when a crew member was wearing the vest is concerning. Within our EMS agency’s EMR, patient race and ethnicity compose a combined category and are documented by the crew. Although clinicians are encouraged to inquire from their patients their preferred race and ethnicity, some have anecdotally described situations in which this is determined and coded by the clinician rather than the patient. This practice may lead to mischaracterization of a patient’s true demographics. Additionally, given that this documentation feature was optional, it was unreported and therefore unknown in nearly one-third of all runs during our study period; however, a sensitivity analysis excluding this unknown race and ethnicity group consistently demonstrated a significant increase in patients declining treatment and/or transport from EMS crews wearing vests. Likewise, EMS crews interpreted and self-reported the violence through the EMR checkbox. Unconscious bias may make it more likely that racial and ethnic minorities are perceived as violent, which has been proposed in previous studies.^33,34,35^ Despite these limitations, the data available to us point toward an increased rate of treatment and/or transport declines by minority patients cared for by crews with at least 1 vested crewmember. Our findings may be attributable to our vested EMS clinicians sharing an appearance with that of law enforcement with no identifiable medical markings on the vest other than the staff member’s name tag indicating their credentials. Prominent agency patches on the underlying white uniform shirt are not readily visible from the front or back. Again, further research is needed to determine the patient-centered perspective for our findings as well as evaluation of concordance of race or ethnicity between patients and crew members; however, EMS clinician race and/or ethnicity was not available for this study. We found crews with less years’ experience within the agency were associated with higher WPV, as were all-female crews. It is possible that more experienced crews may be better at de-escalation techniques. All-female crews may have experienced increased rates of sexual harassment and/or sexual assault, which we did not delineate in the WPV checkbox from verbal abuse and physical assault, respectively.

The violence EMS clinicians experience in the US on 2% to 5% of all prehospital patient encounters has primarily involved verbal abuse and physical assault in the absence of penetrating injuries.^3,5,7,36,37^ Data from the BLS identified more than 14 000 (67%) injuries reported by emergency medical technicians and paramedics involved sprains or strains including back injury (43%), compared with 530 assaults, 5 of which were fatal.^38^ A survey study of EMS clinicians in 2013 revealed an overall prevalence of violence of 69% among respondents over the prior 12 months, with 35 respondents (2%) indicating an actual or attempted stabbing and 21 (1%) indicating an actual or attempted shooting.^7^ More recent data from the BLS identified 350 (22%) of all EMS violence-related injuries between 2012 and 2015 involved the trunk, where body armor may have offered some degree of protection; additionally, 300 injuries (18%) were classified as occurring to multiple body parts, and may have also included the trunk and have been lessened by vest wear.^39^ Although violence enacted against EMS with weapons is not as common as other types of violence perpetrated, the higher probability of severe injury or loss of life of any EMS clinician attributable to this high-threat violence is one loss too many and the impact is often felt throughout the entire first responder community.

The authors of this study do not collectively advocate for or against body armor use by EMS clinicians, but rather seek to present comprehensive and previously unknown considerations for agency leadership. The safety of EMS clinicians on the job is of the utmost importance; however, threats to prehospital personnel include musculoskeletal and overuse injuries, in addition to violence. Patient-centered care is additionally a cornerstone of prehospital practice and risks of further isolating historically marginalized patient populations from health care should be carefully considered when weighing all benefits and risks related to body armor usage by EMS.

### Limitations

This study has several important limitations in addition to those already discussed. Although implementation of a WPV checkbox integrated within the EMR improved our ability to capture violent events across our agency by 30-fold,^40^ we acknowledge additional incidents of WPV remain unrecognized and/or unreported by EMS clinicians due to longstanding barriers that have been described elsewhere.^41,42^ Impressions of EMS clinicians may not necessarily be accurate, such as indicating psychiatric or behavioral impressions for the majority of violent patient encounters that may have been related to underlying medical conditions causing severe agitation. Certain EMS clinician demographics such as age, race, and ethnicity were not available for analysis. Additionally, although our large multistate ambulance agency is diverse and encompasses both rural and urban settings, our findings are isolated to 1 single EMS agency in the Midwest and may not be representative of other agencies.

## Conclusions

In this cohort study, vested crews experienced increased prevalence of WPV compared with nonvested crews. Use of vests increased the frequency of all patients declining EMS treatment and/or transport. Among minority groups, there was a significant increase in Black or African American patients declining treatment and/or transport. Agencies should consider benefits and unintended consequences of EMS clinicians wearing body armor.

## Notes

Supplement 1. **eTable 1.** Frequency of Outcomes Indicating Treatment and/or Transport Declined by Individual Minority Racial/Ethnic Patient Groups Based on EMS Crew Wearing Protective Vests With Risk Ratio **eTable 2. **Prevalence of WPV Experienced per 100 Runs, Preintervention (November 14, 2022 to March 13, 2023), Compared With Postintervention (April 1, 2023 to March 31, 2024) Among EMS staff Who Did and Did Not Opt In to the Vest Intervention

Supplement 2.
Data Sharing Statement

## References

[1] US Bureau of Labor Statistics. Employer-reported workplace injuries and illnesses—2021-2022. 2023. Accessed May 9, 2024. https://www.bls.gov/news.release/pdf/osh.pdf

[2] NAEMT. Violence against EMS practitioners: 2019 national survey. 2019. Accessed May 9, 2024. https://www.naemt.org/docs/default-source/2017-publication-docs/naemt-violence-report-web-10-02-2019.pdf

[3] Murray RM, Davis AL, Shepler LJ, . A systematic review of workplace violence against emergency medical services responders. New Solut. 2020;29(4):487-503. doi:10.1177/1048291119893388 31841060 PMC8594050

[4] Maguire BJ, O’Meara P, O’Neill BJ, Brightwell R. Violence against emergency medical services personnel: a systematic review of the literature. Am J Ind Med. 2018;61(2):167-180. doi:10.1002/ajim.22797 29178541

[5] Grange JT, Corbett SW. Violence against emergency medical services personnel. Prehosp Emerg Care. 2002;6(2):186-190. doi:10.1080/10903120290938526 11962565

[6] Pozzi C. Exposure of prehospital providers to violence and abuse. J Emerg Nurs. 1998;24(4):320-323. doi:10.1016/S0099-1767(98)90104-0 9814235

[7] Gormley MA, Crowe RP, Bentley MA, Levine R. A national description of violence toward emergency medical services personnel. Prehosp Emerg Care. 2016;20(4):439-447. doi:10.3109/10903127.2015.1128029 26836247

[8] Furin M, Eliseo LJ, Langlois B, Fernandez WG, Mitchell P, Dyer KS. Self-reported provider safety in an urban emergency medical system. West J Emerg Med. 2015;16(3):459-464. doi:10.5811/westjem.2015.2.24124 25987930 PMC4427227

[9] National Institute of Justice. Body armor. Accessed May 9, 2024. https://nij.ojp.gov/topics/equipment-and-technology/body-armor

[10] Barszewski L. Fla. firefighter-paramedics getting body armor, helmets. Fire Rescue 1. Accessed May 9, 2024. https://www.firerescue1.com/fire-products/personal-protective-equipment-ppe/articles/fla-firefighter-paramedics-getting-body-armor-helmets-VsVVd6ivs910qA1e/

[11] Danylko R. Bulletproof vests now mandatory for all Cleveland EMS responses. Accessed May 9, 2024. https://www.cleveland.com/metro/2016/01/bulletproof_vests_now_mandatory.html

[12] Abel B. Detroit Fire and EMS now required to wear body armor. WXYZ Detroit. Accessed May 9, 2024. https://www.wxyz.com/news/region/detroit/detroit-fire-and-ems-now-required-to-wear-body-armor

[13] Eckstein M, Cowen AR. Scene safety in the face of automatic weapons fire: a new dilemma for EMS? Prehosp Emerg Care. 1998;2(2):117-122. doi:10.1080/10903129808958854 9709330

[14] Brown LH, Waldman J, Copeland TW, Smithson WE, Prasad NH. Mistaken identity: the effect of badges on EMT recognition. Prehosp Disaster Med. 1995;10(3):195-197. doi:10.1017/S1049023X0004200X 10155429

[15] Spelten E, van Vuuren J, O’Meara P, . Workplace violence against emergency health care workers: what strategies do workers use? BMC Emerg Med. 2022;22(1):78. doi:10.1186/s12873-022-00621-9 35524175 PMC9074314

[16] Levenson E, Campbell J. Shootings of police officers highlight a rise in violence and distrust. CNN. 2022. Accessed May 9, 2024. https://www.cnn.com/2022/10/17/us/police-violence-ambush-attack/index.html

[17] Ponsi L. Support available to help with stress stemming from anti-police sentiment. Behind the Badge. 2020. Accessed May 17, 2024. https://behindthebadge.com/support-available-to-help-with-stress-stemming-from-anti-police-sentiment/

[18] Jany L. Minneapolis-area EMS plans uniform change to distinguish medics from law enforcement. EMS1. Accessed May 9, 2024. https://www.ems1.com/ems-management/articles/minneapolis-area-ems-plans-uniform-change-to-distinguish-medics-from-law-enforcement-PeTPsbAnMQOCW33m/

[19] Atkinson WK and Bassett AW. Dodging bullets. Gang violence and EMS. J EMS Med. 1993;18(7):55-57. 10127348

[20] IACP/Dupont Kevlar Survivor’s Club. Accessed May 9, 2024. https://www.dupont.com.tr/military-law-enforcement-and-emergency-response/the-kevlar-survivors-club.html#:~:text=This%20partnership%20began%20in%201987,request%20with%20academia%2C%20the%20Dept

[21] Liu W, Taylor B. The effect of body armor on saving officers’ lives: An analysis using LEOKA data. J Occup Environ Hyg. 2017;14(2):73-80. doi:10.1080/15459624.2016.1214272 27715652

[22] Jacoby SF, Richmond TS, Holena DN, Kaufman EJ. A safe haven for the injured? Urban trauma care at the intersection of healthcare, law enforcement, and race. Soc Sci Med. 2018;199:115-122. doi:10.1016/j.socscimed.2017.05.037 28552292 PMC5694382

[23] Patton D, Sodhi A, Affinati S, Lee J, Crandall M. Post-discharge needs of victims of gun violence in Chicago: a qualitative study. J Interpers Violence. 2019;34(1):135-155. doi:10.1177/0886260516669545 27638688

[24] Liebschutz J, Schwartz S, Hoyte J, . A chasm between injury and care: experiences of black male victims of violence. J Trauma. 2010;69(6):1372-1378. doi:10.1097/TA.0b013e3181e74fcf 20838259 PMC3005415

[25] von Elm E, Altman DG, Egger M, Pocock SJ, Gøtzsche PC, Vandenbroucke JP; STROBE Initiative. The Strengthening the Reporting of Observational Studies in Epidemiology (STROBE) statement: guidelines for reporting observational studies. Lancet. 2007;370(9596):1453-1457. doi:10.1016/S0140-6736(07)61602-X 18064739

[26] The Joint Commission. Workplace violence prevention resources. Accessed May 9, 2024. https://www.jointcommission.org/resources/patient-safety-topics/workplace-violence-prevention/

[27] Understanding NIJ. 0101.06 armor protection levels. Justice Technology Information Center. Accessed January 15, 2023. https://www.ojp.gov/pdffiles1/nij/nlectc/250144.pdf

[28] Schuman D, Kaplan J, Mohs M, Rantala J. City identifies 2 officers, 1 paramedic killed in Burnsville, Minnesota; suspect also dead. WCCO News. 2024. Accessed May 17, 2024. https://www.cbsnews.com/minnesota/news/burnsville-incident-with-weapons-law-enforcement-responds/

[29] Frehse R, Reilly L, Colbert C. A New York EMT was stabbed to death while on her break in an ‘unprovoked attack’. CNN. 2022. Accessed May 17, 2024. https://www.cnn.com/2022/10/01/us/fdny-emt-stabbed-in-queens/index.html

[30] Worrell G. NYC EMT stabbed eight times by unhinged patient in ambulance speaks out: ‘Thought I was going to die’. New York Post. 2024. Accessed May 17, 2024. https://nypost.com/2024/05/11/us-news/emt-julia-fatum-who-was-stabbed-in-her-ambulance-left-with-nerve-damage-and-nightmares/

[31] Ikeda E. Paramedic stabbed in neck by very person he was trying to help, co-workers say. Fox 5 Atlanta. 2020. Accessed May 17, 2024. https://www.fox5atlanta.com/news/paramedic-stabbed-in-neck-by-very-person-he-was-trying-to-help-co-workers-say

[32] Ritchie DT, Doyle C. Ergonomic impact of prehospital clinicians using body armour: A qualitative study. Appl Ergon. 2023;108:103947. doi:10.1016/j.apergo.2022.103947 36462452

[33] Wong AH, Whitfill T, Ohuabunwa EC, . Association of race/ethnicity and other demographic characteristics with use of physical restraints in the emergency department. JAMA Netw Open. 2021;4(1):e2035241. doi:10.1001/jamanetworkopen.2020.35241 33492372 PMC7835716

[34] Haimovich AD, Taylor RA, Chang-Sing E, . Disparities associated with electronic behavioral alerts for safety and violence concerns in the emergency department. Ann Emerg Med. 2024;83(2):100-107. doi:10.1016/j.annemergmed.2023.04.004 37269262 PMC10689576

[35] Agarwal AK, Seeburger E, O’Neill G, . Prevalence of behavioral flags in the electronic health record among Black and White patients visiting the emergency department. JAMA Netw Open. 2023;6(1):e2251734. doi:10.1001/jamanetworkopen.2022.51734 36656576 PMC9857105

[36] McGuire SS, Bellolio F, Buck B, . Workplace violence against emergency medical services (EMS): a prospective 12-month cohort study evaluating prevalence and risk factors within a large, multistate EMS agency. Prehosp Emerg Care. 2024:1-8. doi:10.1080/10903127.2024.2411020 39356230

[37] Mock EF, Wrenn KD, Wright SW, Eustis TC, Slovis CM. Prospective field study of violence in emergency medical services calls. Ann Emerg Med. 1998;32(1):33-36. doi:10.1016/S0196-0644(98)70096-4 9656946

[38] Maguire BJ, Smith S. Injuries and fatalities among emergency medical technicians and paramedics in the United States. Prehosp Disaster Med. 2013;28(4):376-382. doi:10.1017/S1049023X13003555 23659321

[39] Maguire BJ, O’Neill BJ. Emergency medical service personnel’s risk from violence while serving the community. Am J Public Health. 2017;107(11):1770-1775. doi:10.2105/AJPH.2017.303989 28933934 PMC5637660

[40] McGuire SS, Bellolio F, Buck BJ, Stuhr DD, Buffum MR, Liedl CM. Improving documentation of workplace violence (WPV) experienced by prehospital personnel. PEC; 2023.

[41] McGuire SS, Finley JL, Gazley BF, Mullan AF, Clements CM. Workplace violence reporting behaviors in emergency departments across a health system. Annal Emerg Med. 2021;78(4):S8. doi:10.1016/j.annemergmed.2021.09.026 PMC10047752 36976596

[42] Phillips JP. Workplace violence against health care workers in the United States. N Engl J Med. 2016;374(17):1661-1669. doi:10.1056/NEJMra1501998 27119238

