# Supplementary material for: Use of Body Armor by EMS Clinicians, Workplace Violence, and Racial and Ethnic Disparities in Care
Source: JAMA Netw Open. 2025 Jan 29;8(1):e2456528. doi: 10.1001/jamanetworkopen.2024.56528 (PMC11780475; doi:10.1001/jamanetworkopen.2024.56528)
Supplement: Supplement 1. — eTable 1. Frequency of Outcomes Indicating Treatment and/or Transport Declined by Individual Minority Racial/Ethnic Patient Groups Based on EMS Crew Wearing Protective Vests With Risk Ratio eTable 2. Prevalence of WPV Experienced per 100 Runs, Preintervention (November 14, 2022 to March 13, 2023), Compared With Postintervention (April 1, 2023 to March 31, 2024) Among EMS staff Who Did and Did Not Opt In to the Vest Intervention [file jamanetwopen-e2456528-s001.pdf]

Supplemental Online Content

McGuire SS, Bellolio F, Sztajnkrycer MD, et al. Emergency medical services body armor, workplace violence, and racial disparities in care. *JAMA Netw Open*. 2025;8(1):e2456528. doi:10.1001/jamanetworkopen.2024.56528

eTable 1. Frequency of Outcomes Indicating Treatment and/or Transport Declined by Individual Minority Racial/Ethnic Patient Groups Based on EMS Crew Wearing Protective Vests With Risk Ratio
eTable 2. Prevalence of WPV Experienced per 100 Runs, Preintervention (November 14, 2022 to March 13, 2023), Compared With Postintervention (April 1, 2023 to March 31, 2024) Among EMS staff Who Did and Did Not Opt In to the Vest Intervention

This supplemental material has been provided by the authors to give readers additional information about their work.

© 2024 McGuire SS et al. *JAMA Network Open*.

eTable 1. Frequency of Outcomes Indicating Treatment and/or Transport Declined by Individual Minority Racial/Ethnic Patient Groups Based on EMS Crew Wearing Protective Vests With Risk Ratio

EMS Run Outcome	No Members Vested	≥1 Member(s) Vested	Unadjusted Risk Ratio (95% CI)	P-Value
**American Indian/ Alaskan Native**	N = 407	N = 464		
Declined treatment/transport	30 (7.4%)	41 (8.8%)	1.17 (0.73, 1.87)	0.50
Did not decline treatment/transport	377 (92.6%)	423 (91.2%)		
**Asian**	N = 243	N = 269		
Declined treatment/transport	31 (12.8%)	45 (16.7%)	1.24 (0.80, 1.93)	0.34
Did not decline treatment/transport	212 (87.2%)	224 (83.3%)		
**Black/African American**	N = 1626	N = 2618		
Declined treatment/transport	223 (13.7%)	461 (17.6%)	1.28 (1.10, 1.49)	0.002
Did not decline treatment/transport	1403 (86.3%)	2157 (82.4%)		
**Hispanic or Latino**	N = 553	N = 805		
Declined treatment/transport	107 (19.3%)	155 (19.3%)	1.01 (0.80, 1.28)	0.93
Did not decline treatment/transport	446 (80.7%)	650 (80.7%)		
**Native Hawaiian/ Pacific Islander**	N = 70	N = 72		
Declined treatment/transport	8 (11.4%)	6 (8.3%)	0.72 (0.26, 1.96)	0.52
Did not decline treatment/transport	62 (88.6%)	66 (91.7%)		

© 2024 McGuire SS et al. *JAMA Network Open*.

eTable 2. Prevalence of WPV Experienced per 100 Runs, Preintervention (November 14, 2022 to March 13, 2023), Compared With Postintervention (April 1, 2023 to March 31, 2024) Among EMS staff Who Did and Did Not Opt In to the Vest Intervention

	**Staff who did not opt in to the vest program**			**Staff who opted in to the vest program**		
	**Pre-Intervention** (N = 53972)	**Post-Intervention** (N = 176430)	**P-Value**	**Pre-Intervention** (N = 19050)	**Post-Intervention** (N = 51408)	**P-Value**
Any violence	0.97 (0.89, 1.06)	0.96 (0.91, 1.00)	0.77	1.12 (0.98, 1.28)	1.10 (1.02, 1.20)	0.84
Verbal abuse	0.80 (0.72, 0.88)	0.79 (0.75, 0.83)	0.85	0.90 (0.77, 1.05)	0.89 (0.81, 0.97)	0.86
Physical assault	0.39 (0.34, 0.45)	0.43 (0.40, 0.47)	0.15	0.46 (0.37, 0.57)	0.48 (0.42, 0.54)	0.77

© 2024 McGuire SS et al. *JAMA Network Open*.
