# Supplementary material for: Use of Body Armor by EMS Clinicians, Workplace Violence, and Racial and Ethnic Disparities in Care
Source: JAMA Netw Open. 2025 Jan 29;8(1):e2456528. doi: 10.1001/jamanetworkopen.2024.56528 (PMC11780475; doi:10.1001/jamanetworkopen.2024.56528)
Supplement: Supplement 2. — Data Sharing Statement [file jamanetwopen-e2456528-s002.pdf]

Data Sharing Statement

McGuire. Emergency Medical Services Body Armor, Workplace Violence, and Racial Disparities in Care. *JAMA Netw Open*. Published January 27, 2025.
doi:10.1001/jamanetworkopen.2024.56528

Data
Data available: Yes
Data types: Deidentified participant data
How to access data: De-identified datasets used and analyzed in the study are available from the corresponding author (mcguire.sarayna@mayo.edu) on reasonable request.
When available: With publication

Supporting Documents
Document types: None

Additional Information
Who can access the data: De-identified datasets used and analyzed in the study are available from the corresponding author (mcguire.sarayna@mayo.edu) on reasonable request.
Types of analyses: on reasonable request
Mechanisms of data availability: n/a
